# Comparing Conventional Chemotherapy to Chronomodulated Chemotherapy for Cancer Treatment: Protocol for a Systematic Review

**DOI:** 10.2196/18023

**Published:** 2020-10-21

**Authors:** Aoife B Kilgallen, Urška Štibler, Markella I Printezi, Marrit Putker, Cornelis J A Punt, Joost P G Sluijter, Anne M May, Linda W van Laake

**Affiliations:** 1 Regenerative Medicine Centre University Medical Centre Utrecht Utrecht Netherlands; 2 Division Heart and Lungs University Medical Centre Utrecht Utrecht Netherlands; 3 Hubrecht Institute, Royal Netherlands Academy of Arts and Sciences (KNAW) University Medical Centre Utrecht Utrecht Netherlands; 4 Julius Centre for Health Sciences and Primary Care University Medical Centre Utrecht Utrecht University Utrecht Netherlands; 5 Experimental Cardiology Laboratory University Medical Centre Utrecht Utrecht Netherlands; 6 Circulatory Health Laboratory University Medical Centre Utrecht Utrecht Netherlands; 7 University Utrecht Utrecht Netherlands

**Keywords:** cancer, chemotherapy, chronotherapy, circadian clock, efficacy, overall survival, safety, systematic review

## Abstract

**Background:**

Chronomodulated chemotherapy aims to achieve maximum drug safety and efficacy by adjusting the time of treatment to an optimal biological time as determined by the circadian clock. Although it is a promising alternative to conventional (non–time-stipulated) chemotherapy in several instances, the lack of scientific consensus and the increased logistical burden of timed administration limit the use of a chronomodulated administration protocol.

**Objective:**

With the goal to increase scientific consensus on this subject, we plan to conduct a systematic review of the current literature to compare the drug safety and efficacy of chronomodulated chemotherapy with those of conventional chemotherapy.

**Methods:**

This systematic review will comply with the PRISMA (Preferred Reporting Items for the Systematic Reviews and Meta-Analysis) guidelines. In order to identify relevant studies, we conducted a comprehensive search in PubMed and Embase on May 18, 2020. We included clinical studies that compare either the safety or efficacy of chronomodulated chemotherapy with that of conventional chemotherapy. Potential studies will be reviewed and screened by 2 independent reviewers. Quality assessment will be performed using the National Institutes of Health’s Study Quality Assessment Tool (Quality Assessment of Controlled Intervention Studies). Disagreements will be resolved by consulting a third independent reviewer.

**Results:**

This protocol has received funding, and the search for studies from databases commenced on May 18, 2020. The systematic review is planned to be completed by October 31, 2020.

**Conclusions:**

In this systematic review, we will compare drug safety and drug efficacy for cancer patients who were administered either chronomodulated chemotherapy or conventional chemotherapy. Moreover, we will highlight the outcomes and quality of the selected trials for this review.

**Trial Registration:**

PROSPERO International Prospective Register of Systematic Reviews CRD42020177878; https://tinyurl.com/y53w9nq6

**International Registered Report Identifier (IRRID):**

PRR1-10.2196/18023

## Introduction

Circadian rhythms (circa: about, dia: a day) organize biological functions in living organisms around an approximate 24-hour period to adjust organ and tissue physiology to the ever-changing demands of day and night [1]. Controlled by a molecular clockwork, they drive oscillations in a broad range of biological processes, ranging from the cellular level (eg, cell cycle regulation [2] and cellular metabolism [3]) to whole body physiology (eg, liver and renal activity [4]), which can therefore greatly affect drug responses [5].

Chemotherapy is used to treat many cancers and involves administration of cytotoxic drugs that either kill or interfere with the proliferation of rapidly dividing cells. The principle of conventional chemotherapy is to increase the chemotherapy dose until maximum cytotoxicity occurs and a maximum tolerated dose is reached. However, as both malignant and normal cells are affected, severe toxicities are often developed, in turn leading to interruption of chemotherapy treatments and decreased survival rates [6]. Therefore, finding ways to increase efficacy and reduce side effects would greatly improve cancer therapy potential.

The molecular mechanisms involved in regulating pharmacological processes such as drug absorption, distribution, metabolism, and excretion are controlled by the circadian clock. The clock thereby determines when the anticancer drug treatment should ideally be delivered as it controls the drug’s elimination and detoxification, affecting the efficacy and toxicity on tumor cells and healthy cells, respectively [7]. In addition to this, the sensitivity of molecular drug targets shows diurnal variations [7]. The tolerability of more than 40 anticancer drugs such as oxaliplatin have demonstrated a 10-fold variability in rodents as a function of dosing time [7]. Anticancer drugs have shown to have a 24-hour variability in drug toxicity as well as improved efficacy and tolerability in rodents kept in alternate exposure to 12h light and 12h darkness (LD12:12) [7]. The circadian rhythms of both drug toxicity and efficacy are defined in preclinical studies, and the optimal circadian time from rodents is extrapolated and translated to the most suitable time to administer chemotherapy according to the human’s circadian rhythms [8]. Together, these findings have led to the investigation of the time-dependent changes in the efficacy and safety of anticancer drug therapies [7].

Chronomodulated chemotherapy aims to exploit the circadian variation in drug response by administering anticancer drugs at specific times of the day, thereby hitting cancer cells when they are most vulnerable or normal cells when they are least vulnerable [9,10]. The terms chronomodulated and circadian-based have been used to describe similar strategies using parenteral or oral administration. For the sake of clarity, we will use the term chronomodulation for all therapeutic routes.

The goal of chronomodulated chemotherapy is to minimize toxic side effects while promoting the maximum achievable efficacy of the chemotherapy regimen to improve the cancer patients’ quality of life, survival time that is based on the different circadian rhythms of DNA synthesis, and cell growth between tumor and normal cells [11,12]. The impact of circadian rhythms on the outcome of cancer therapy has been the subject of several clinical trials over the past two decades. While conventional chemotherapy generally consists of constant-rate drug delivery, chronomodulated chemotherapy is administered as a variable rate infusion with peak drug delivery times set to vary according to circadian time or delivery restricted to specific time windows [13].

To date, several studies have established the association between circadian disruption caused by shiftwork and increased cancer risk [14-17]. In 2007, the International Agency for Research on Cancer classified “shiftwork that involves circadian disruption” as a probable carcinogenic risk [18]. Furthermore, both cancer and chemotherapy have been found to disturb circadian rhythmicity independently of each other [19]. These factors may be causing interpatient variability of circadian rhythms to a yet unknown extent. This leaves room for the exploration of optimizing chronotherapy by adjusting administration time to each patient’s individual circadian clock.

Although chronomodulated chemotherapy is a promising avenue of research that could contribute significantly to improve existing and future cancer treatments, no medical consensus has been reached to implement chronomodulated chemotherapy regimens, and conventional chemotherapy is most often administered in accordance with the hospital schedule and staff working hours [10]. Before this approach is integrated into clinical practice, its benefits must be adequately supported with evidence from well-designed randomized clinical trials.

This systematic review will evaluate the findings of randomized controlled trials (RCTs) that compare the safety and efficacy of chronomodulated chemotherapy with those of conventional chemotherapy administration in adult cancer patients.

## Methods

The systematic review and its protocol will comply with the PRISMA (Preferred Reporting Items for Systematic Reviews and Meta-Analysis) guidelines [20].

### Inclusion and Exclusion Criteria

Studies in English, Dutch, or German will be included in the systematic review. Clinical trials that compare the safety or efficacy of chemotherapy administered to adult cancer patients in accordance with conventional and chronomodulated chemotherapy regimens will be included. Only RCTs will be included in the systematic review. There will be no restrictions on inclusion of studies by time frame or type of setting. We will provide a list of all excluded clinical trials as a supplemental file.

### Participants

Adult patients (≥18 years old) of all ethnicities and genders that are diagnosed with any type of cancer will be included. No restrictions will be imposed with respect to participants having received other treatment prior to inclusion in the trial.

### Interventions

RCTs that compare the clinical safety or efficacy of chemotherapy administered according to circadian delivery schedules with standard delivery schedules will be included. Single agent and combination chronotherapy regimens delivered through oral administration, bolus injection, or infused according to a flat or sinusoidally chronomodulated schedule will be included. In addition, trials with concomitant radiotherapy will be included in this systematic review.

### Outcome Measures

The outcome of this systematic review will be analyzed as per the following measures.

#### Safety

This will include changes in chemotherapy administration, like a delay or a reduction of chemotherapy treatment, maximum tolerable dose, termination of treatment, or early withdrawal from the clinical trial. Toxicity will be assessed by either of the following toxicity grading scales: World Health Organization standard toxicity criteria [21], Common Toxicity Criteria [22], Eastern Cooperation Oncology Group criteria for toxicity [23], Radiation Therapy Oncology Group criteria [24], and Gynecologic Oncology Group standard toxicity criteria.

#### Efficacy

This will include objective response rate (also known as response rate), disease control rates, progression free survival, and overall survival, taking into account salvage therapies if reported. In the case of neoadjuvant and adjuvant studies, efficacy will be measured by recurrence rate and disease-free and overall survival.

### Search Strategy

Studies published on or before May 18, 2020, were identified by performing a comprehensive search in PubMed and Embase (Elsevier). A literature search strategy was developed in collaboration with an information specialist from Utrecht University unconnected to this study. The search strategy was composed of the following terms and their synonyms: cancer, circadian rhythms, and chemotherapy. For chemotherapy, alongside its synonyms, specific terms for chemotherapy classes, individual drug names, and brand names were included. The World Health Organization’s list of essential medicines 2019 [25] was utilized to confirm inclusion of all essential chemotherapies. Appropriate MeSH (Medical Subject Headings) and Embase subjects headings (Emtree terms) were added to the search strategy for PubMed and Embase searches, respectively. No limits on publication dates were imposed on the search. The search results were narrowed down to show only articles in English, Dutch, or German, due to resource limits. For Embase, conference abstracts were filtered from the search results. The complete search is listed in Multimedia Appendix 1. The reference lists of all selected articles were handsearched, and their titles and abstracts will be assessed based on the inclusion and exclusion criteria.

### Study Selection

The appropriate references and trials for the review will be deduplicated in Endnote (Clarivate Analytics) and afterwards uploaded to Rayyan [26] to be screened on title and abstract and will be reviewed independently by 2 different reviewers (ABK and MIP). Disagreements will be resolved by consulting a third independent reviewer (LWL). Full texts that contain studies that could be suitable for the systematic review will be screened based on the inclusion and exclusion criteria. If there is no full text available for a relevant trial, the corresponding author(s) will be contacted in order to request it.

### Risk of Bias and Quality Assessment

The quality and the risk of bias for selected trials and studies will be assessed using the Quality Assessment of Controlled Intervention Studies tool from the National Heart, Lung, and Blood Institute [27]. All included studies will be categorized as being of good, fair, or poor quality. Studies judged as being of poor quality will be excluded from this review. The risk of bias and quality assessment will be performed by 2 reviewers (ABK and MIP). Any inconsistencies between the 2 reviewers will be resolved by consulting a third reviewer (LWL).

Selective reporting within studies will be assessed by verifying if a protocol was published for each included study and evaluating whether all specified outcomes were published. Other possible risks of meta-bias such as publication bias will be discussed appropriately.

The confidence in the cumulative evidence obtained will be assessed using GRADE (Grading of Recommendations, Assessment, Development and Evaluations) [28] for each primary outcome measure.

### Data Extraction

A data extraction sheet will be developed to store information about the selected studies and trials. Each paper will be assessed independently by 2 reviewers (MIP and ABK) to reduce bias. Data from the selected studies and trials will be extracted independently by the 2 reviewers. The data extraction form will be made and piloted before final implementation by both reviewers. The following parameters will be extracted:

1. Publication information: authors, year, country, and journal.
2. Study information: trial size, subject characteristics, type of cancer, duration and frequency of follow-up, chemotherapy, and trial objectives.
3. Treatment information: type of treatment schedule for each patient.
4. Primary outcome variables: efficacy (objective response rate, overall survival, and progression-free survival); toxicity (incidence of side effects and severity of side effects based on either World Health Organization’s standard toxicity criteria [21] or the National Cancer Institute’s Common Terminology Criteria for Adverse Events [22]).
5. Secondary outcome variables: efficacy (disease control rate, reduction in tumor marker, complete response, pathological complete response, disease-free survival, event-free survival, recurrence rate, reoperation rate, rate of microscopically complete resection, time to progression of cancer symptoms, subjective tumor-related symptoms, minimal residual disease, metastasis-free survival); toxicity (duration of side effect, reversibility of side effect, dose limiting toxicity, treatment modifications, treatment delays, and treatment discontinuations); efficacy and toxicity (quality of life, time to treatment failure).

All factors influencing toxicity and efficacy will be explored appropriately. In order to compare the endpoints in both study arms, we will collect the corresponding hazard ratios, relative risks, risk differences, mean/median differences, and their statistical significance, as far as availability allows.

### Amendments

Any amendments made to the protocol will be documented in PROSPERO (the International Prospective Register of Systematic Reviews) by MIP. The methods section of the systematic review will include a summary of any protocol amendments accompanied by date and rationale.

## Results

The search for the relevant studies on databases was performed on May 18, 2020. The systematic review is intended to be completed and ready for submission for publication by October 31, 2020.

## Discussion

This systematic review will have some limitations that will be discussed appropriately. The exclusion of papers not written in English, Dutch, and German may inhibit the identification of all important findings, and this will be taken into consideration in the discussion of the systematic review. However, the database searches of the appropriate studies will be extensive to ensure the required studies are captured for the systematic review.

We will produce a table with patient characteristics, including gender, age, ethnicity, and different cancer types. In this table, we will also include the type of chemotherapy used in the trial, the dose and half-life of these cytotoxic drugs, any significant differences between the conventional and chronomodulated chemotherapy treatment groups, optimal delivery time to administer the chemotherapeutic drug of choice, and the treatment group that produces the least side effects. In case of a sinusoidal infusion schedule, we will also state the circadian timing system function. The selected studies included in this review will provide information required to conclude whether chemotherapy regimens should be administered in accordance with the circadian clock and highlight the findings and quality of reporting these particular clinical trials. This systematic review will provide vital information on drug safety and drug efficacy in cancer patients administered chronomodulated chemotherapy versus conventional chemotherapy.

## Appendix

Multimedia Appendix 1 Search terms.

## Abbreviations

GRADE: Grading of Recommendations, Assessment, Development and Evaluations
MeSH: Medical Subject Headings
PRISMA: Preferred Reporting Items for Systematic Reviews and Meta-Analysis
PROSPERO: International Prospective Register of Systematic Reviews
RCT: randomized controlled trial
