# Supplementary material for: Comparing Conventional Chemotherapy to Chronomodulated Chemotherapy for Cancer Treatment: Protocol for a Systematic Review
Source: JMIR Res Protoc. 2020 Oct 21;9(10):e18023. doi: 10.2196/18023 (PMC7641776; doi:10.2196/18023)
Supplement: Multimedia Appendix 1 [file resprot_v9i10e18023_app1.pdf]

Multimedia Appendix 1: Search Terms

PubMed search terms:

(((((Cancer*[TIAB] OR Malignan*[TIAB] OR Tumor[TIAB] OR Tumors[TIAB] OR Tumorigen*[TIAB] OR Tumour*[TIAB] OR Neoplasm*[TIAB] OR Leukemia*[TIAB] OR Leukaemia*[TIAB] OR Lymphoma*[TIAB] OR Anaplas*[TIAB] OR Carcinogen*[TIAB] OR Carcinom*[TIAB] OR Sarcom*[TIAB] OR Melanom*[TIAB] OR Retinoblastoma*[TIAB] OR Nefroblastoma*[TIAB] OR Neuroblastoma*[TIAB] OR Mesothelioma*[TIAB] OR Medulloblastoma*[TIAB] OR Meningioma*[TIAB] OR Glioma*[TIAB] OR Astrocytoma*[TIAB] OR Glioblastoma*[TIAB] OR Oligodendroglioma*[TIAB] OR Ependymoma*[TIAB])) OR "Neoplasms"[Mesh])) AND (((Chemotherap*[TIAB] OR Chemo therap*[TIAB] OR chronochemotherap*[TIAB] OR Cytostatic*[TIAB] OR Antineoplastic*[TIAB] OR Anti neoplastic*[TIAB] OR Oncolytic*[TIAB] OR Anticancer[TIAB] OR Antitumor[TIAB] OR Antitumour[TIAB] OR Anti tumour[TIAB] OR Anti tumor[TIAB] OR Cytotoxic*[TIAB] OR Anticarcinogenic*[TIAB] OR Anti carcinogenic*[TIAB] OR Anthracyclin*[TIAB] OR Antracyclin*[TIAB] OR Anthracenedion*[TIAB] OR Idarubicin*[TIAB] OR Zavedos[TIAB] OR Demethoxydaunorubicin*[TIAB] OR Demethoxydaunomycin*[TIAB] OR Mitoxantron*[TIAB] OR Ebewe[TIAB] OR Mitoxanthron*[TIAB] OR Pixantron*[TIAB] OR Pixuvri[TIAB] OR Daunorubicin*[TIAB] OR Cerubidin*[TIAB] OR Daunoxome[TIAB] OR leukaemomycin*[TIAB] OR Rubidomycin*[TIAB] OR Rubomycin*[TIAB] OR Daunomycin*[TIAB] OR Acetyladriamycin*[TIAB] OR Doxorubicin*[TIAB] OR Adriamycin*[TIAB] OR Caelyx[TIAB] OR Adriablastin*[TIAB] OR Adriblastin*[TIAB] OR Doxil[TIAB] OR Myocet[TIAB] OR Dactinomycin*[TIAB] OR Cosmegen[TIAB] OR Actinomycin*[TIAB] OR Bleomycin*[TIAB] OR Bleomedac[TIAB] OR Bleocin*[TIAB] OR Bleomicin*[TIAB] OR Mitomycin*[TIAB] OR Kyowa[TIAB] OR Mitocin*[TIAB] OR Mitosol[TIAB] OR Mitozytrex[TIAB] OR Mutamycin*[TIAB] OR Mytomycin*[TIAB] OR Mytozytrex[TIAB] OR Antimetabolite*[TIAB] OR Anti metabolite*[TIAB] OR Purinederivative*[TIAB] OR Purine derivative*[TIAB] OR Purine analog*[TIAB] OR Purine antagonist*[TIAB] OR cladribin*[TIAB] OR Leustatin[TIAB] OR Chlorodeoxyadenosin*[TIAB] OR Litak[TIAB] OR Cladarabin*[TIAB] OR clofarabin*[TIAB] OR Evoltra[TIAB] OR Clolar[TIAB] OR Clofarex[TIAB] OR Fludarabin*[TIAB] OR Fludara[TIAB] OR Mercaptopurin*[TIAB] OR Xaluprin*[TIAB] OR Purinethol*[TIAB] OR Merkaptopurin*[TIAB] OR Merkaptopuryn*[TIAB] OR Hypoxanthin*[TIAB] OR Ismipur[TIAB] OR Leukerin*[TIAB] OR Leupurin*[TIAB] OR Mercaleukin*[TIAB] OR Purixan[TIAB] OR Nelarabin*[TIAB] OR Atriance[TIAB] OR Arranon[TIAB] OR Tioguanin*[TIAB] OR Lanvis[TIAB] OR Thiosix[TIAB] OR Thioguanin*[TIAB] OR Pyrimidine antagonist*[TIAB] OR Pyrimidine analog*[TIAB] OR Fluorouracil*[TIAB] OR Adrucil[TIAB] OR Efudix[TIAB] OR Arumel[TIAB] OR Carac[TIAB] OR Carzonal[TIAB] OR Fluoruracil*[TIAB] OR azacitidin*[TIAB] OR Vidaza[TIAB] OR Azacytidin*[TIAB] OR Ladakamycin*[TIAB] OR Mylosar[TIAB] OR capecitabin*[TIAB] OR Xeloda[TIAB] OR Xabine[TIAB] OR Cytarabin*[TIAB] OR Depocyt*[TIAB] OR Citarabin*[TIAB] OR Alexan[TIAB] OR Arabitin*[TIAB] OR Aracyt*[TIAB] OR Erpalfa[TIAB] OR Tarabin*[TIAB] OR Udicil[TIAB] OR Decitabin*[TIAB] OR Dacogen[TIAB] OR Gemcitabin*[TIAB] OR Zefei[TIAB] OR Gemcel[TIAB] OR Gemlip[TIAB] OR Tegafur[TIAB] OR Teysuno[TIAB] OR Gimeracil[TIAB] OR Oteracil[TIAB] OR Tipiracil[TIAB] OR Lonsurf[TIAB] OR Trifluridin*[TIAB] OR Floxuridin*[TIAB] OR Deoxyuridin*[TIAB] OR Alkylating agent*[TIAB] OR Busulfan*[TIAB] OR Busilvex[TIAB] OR Busulfex[TIAB] OR Myleran[TIAB] OR Bendamustin*[TIAB] OR Bendeka[TIAB] OR Levact[TIAB] OR Carmustin*[TIAB] OR Consium[TIAB] OR Gliadel[TIAB] OR Nitrumon[TIAB] OR Chlorambucil*[TIAB] OR Leukeran[TIAB] OR Clorambucil*[TIAB] OR Chlormethin*[TIAB] OR Ledaga[TIAB] OR Mechlorethamin*[TIAB] OR Mustargen[TIAB] OR Cyclophosphami*[TIAB] OR Endoxan[TIAB] OR Cyclofosfami*[TIAB] OR Cytophospha*[TIAB] OR Dacarbazin*[TIAB] OR Imidazole carboxamide[TIAB] OR Deticene[TIAB] OR Iphosphamide[TIAB] OR Holoxan[TIAB] OR Ifosfami*[TIAB] OR Lomustin*[TIAB] OR Belustin*[TIAB] OR Ceenu[TIAB] OR Gleostine[TIAB] OR Melphalan*[TIAB] OR Alkeran[TIAB] OR Melfalan*[TIAB] OR Evomela[TIAB] OR Procarbazin*[TIAB] OR Natulan[TIAB] OR Matulane[TIAB] OR Indicarb[TIAB] OR Temozolomid*[TIAB] OR Temodal[TIAB] OR Temodar[TIAB] OR Temcad[TIAB] OR Methazolastone[TIAB] OR Thiotepa[TIAB] OR Tepadina[TIAB] OR Tiotepa[TIAB] OR

Treosulfan*[TIAB] OR Trecondi[TIAB] OR Treosulphan*[TIAB] OR Ovastat[TIAB] OR Microtubule inhibitor*[TIAB] OR Taxoid*[TIAB] OR Taxan*[TIAB] OR Docetax*[TIAB] OR Taxotere[TIAB] OR Taxoel[TIAB] OR Cabazitax*[TIAB] OR Jevtana[TIAB] OR Paclitaxel[TIAB] OR Abraxane[TIAB] OR Paclitaxin[TIAB] OR Taxol[TIAB] OR Capxol[TIAB] OR Vinca alkaloid*[TIAB] OR Vincristin*[TIAB] OR Leurocristine[TIAB] OR Oncovin*[TIAB] OR Vincasar[TIAB] OR Citomid[TIAB] OR Vinblastin*[TIAB] OR Velban[TIAB] OR Vincaleukoblastin*[TIAB] OR Vincaleucoblastin*[TIAB] OR Vinorelbin*[TIAB] OR Navelbine[TIAB] OR Exelbine[TIAB] OR Asparaginase[TIAB] OR Erwinase[TIAB] OR Spectrila[TIAB] OR Elspar[TIAB] OR Crisataspase[TIAB] OR Colaspase[TIAB] OR Cisplatin*[TIAB] OR Platinol[TIAB] OR Platamin[TIAB] OR Neoplatin[TIAB] OR Cismaplat[TIAB] OR diamminedichloridoplatin*[TIAB] OR Oxaliplatin*[TIAB] OR Eloxatin[TIAB] OR Oxalisin[TIAB] OR Carboplatin*[TIAB] OR Carbosin[TIAB] OR Paraplatin[TIAB] OR Topoisomerase inhibitor*[TIAB] OR ''Topoisomerase I inhibitor''[TIAB] OR ''Topoisomerase II inhibitor''[TIAB] OR Topoisomerase inhibitors[TIAB] OR ''Topoisomerase I inhibitors''[TIAB] OR ''Topoisomerase II inhibitors''[TIAB] OR Etoposid*[TIAB] OR Etopophos[TIAB] OR Toposar[TIAB] OR Eposin[TIAB] OR Toposin[TIAB] OR Vepesid[TIAB] OR Irinotecan*[TIAB] OR Camptosar[TIAB] OR Campto[TIAB] OR Onivyde[TIAB] OR Irinophore[TIAB] OR Topotecan*[TIAB] OR Hycamtin[TIAB] OR Hycamptamine[TIAB] OR Nogitecan[TIAB] OR Arsenic trioxide[TIAB] OR Trisenox[TIAB] OR Hydroxycarbamid*[TIAB] OR Droxia[TIAB] OR Hydrea[TIAB] OR Hydroxyurea[TIAB] OR Carbamazepin*[TIAB] OR Methotrexate[TIAB] OR Emthexate[TIAB] OR Trexall[TIAB] OR Rheumatrex[TIAB] OR Pegaspargas*[TIAB] OR Oncaspar[TIAB] OR Realgar[TIAB] OR Huangdai[TIAB])) OR ("Consolidation Chemotherapy"[Mesh] OR "Induction Chemotherapy"[Mesh] OR "Maintenance Chemotherapy"[Mesh] OR "Chemotherapy, Adjuvant"[Mesh] OR "Chemotherapy, Cancer, Regional Perfusion"[Mesh] OR "Antineoplastic Combined Chemotherapy Protocols"[Mesh] OR "Anthracyclines"[Mesh] OR "Antibiotics, Antineoplastic"[Mesh] OR "Bleomycin"[Mesh] OR "Mitomycin"[Mesh] OR "Antimetabolites, Antineoplastic"[Mesh] OR "Cladribine"[Mesh] AND "Clofarabine"[Mesh] OR "fludarabine phosphate"[Supplementary Concept] OR "fludarabine"[Supplementary Concept] OR "Mercaptopurine"[Mesh] OR "nelarabine"[Supplementary Concept] OR "Thioguanine"[Mesh] OR "Fluorouracil"[Mesh] OR "Azacitidine"[Mesh] AND "Cytarabine"[Mesh] OR "gemcitabine"[Supplementary Concept] OR "tipiracil"[Supplementary Concept] OR "Deoxyuridine"[Mesh] OR "Antineoplastic Agents, Alkylating"[Mesh] OR "Busulfan"[Mesh] OR "Bendamustine Hydrochloride"[Mesh] OR "Carmustine"[Mesh] OR "Chlorambucil"[Mesh] OR "Mechlorethamine"[Mesh] OR "Cyclophosphamide"[Mesh] OR "Dacarbazine"[Mesh] OR "Lomustine"[Mesh] OR "Melphalan"[Mesh] OR "Procarbazine"[Mesh] OR "Thiotepa"[Mesh] OR "treosulfan"[Supplementary Concept] OR "Antimitotic Agents"[Mesh] OR "Taxoids"[Mesh] OR "cabazitaxel"[Supplementary Concept] OR "Vinca Alkaloids"[Mesh] OR "Asparaginase"[Mesh] OR "Cisplatin"[Mesh] OR "Oxaliplatin"[Mesh] OR "Carboplatin"[Mesh] OR "Topoisomerase Inhibitors"[Mesh] OR "Etoposide"[Mesh] OR "Irinotecan"[Mesh] OR "Topotecan"[Mesh] OR "Arsenic Trioxide"[Mesh] OR "Hydroxyurea"[Mesh] OR "Methotrexate"[Mesh] OR "pegaspargase"[Supplementary Concept] OR "realgar-indigo naturalis"[Supplementary Concept]))) AND (((Circadian[TIAB] OR Clock*[TIAB] OR Biological rhythm[TIAB] OR Biological rhythms[TIAB] OR Chronomodulat*[TIAB] OR Chrono modulated[TIAB] OR Chrono modulate[TIAB] OR Chrono modulation[TIAB] OR Chronotherap*[TIAB] OR Chrono therap*[TIAB] OR Chrono therapeutic*[TIAB] OR Chronobiolog*[TIAB] OR Chrono biology[TIAB] OR Chrono biological[TIAB] OR Nycthemeral[TIAB] OR Nyctohemeral[TIAB] OR Diurnal[TIAB] OR chronochemotherap*[TIAB])) OR ("Chronotherapy"[Mesh] OR "Chronobiology Phenomena"[Mesh]))

EMBASE (Elsevier) search terms:

((cancer*:ti,ab,kw OR malignan*:ti,ab,kw OR tumor*:ti,ab,kw OR tumour*:ti,ab,kw OR neoplasm*:ti,ab,kw OR leukemia*:ti,ab,kw OR leukaemia*:ti,ab,kw OR lymphoma*:ti,ab,kw OR anaplas*:ti,ab,kw OR carcinogen*:ti,ab,kw OR carcinom*:ti,ab,kw OR sarcom*:ti,ab,kw OR melanom*:ti,ab,kw OR retinoblastoma*:ti,ab,kw OR nefroblastoma*:ti,ab,kw OR neuroblastoma*:ti,ab,kw OR mesothelioma*:ti,ab,kw OR medulloblastoma*:ti,ab,kw OR meningioma*:ti,ab,kw OR glioma*:ti,ab,kw OR astrocytoma*:ti,ab,kw OR glioblastoma*:ti,ab,kw OR oligodendroglioma*:ti,ab,kw OR ependymoma*:ti,ab,kw OR 'neoplasm'/exp) AND (chemotherap*:ti,ab,kw OR 'chemo therap*':ti,ab,kw OR chronochemotherap*:ti,ab,kw OR cytostatic*:ti,ab,kw OR antineoplastic*:ti,ab,kw OR 'anti neoplastic*':ti,ab,kw OR oncolytic*:ti,ab,kw OR anticancer:ti,ab,kw OR antitumor:ti,ab,kw OR 'anti tumor':ti,ab,kw OR 'antitumour':ti,ab,kw OR 'anti tumour':ti,ab,kw OR cytotoxic*:ti,ab,kw OR anticarcinogenic*:ti,ab,kw OR 'anti carcinogenic*':ti,ab,kw OR anthracyclin*:ti,ab,kw OR antracyclin*:ti,ab,kw OR anthracenedion*:ti,ab,kw OR idarubicin*:ti,ab,kw OR zavedos:ti,ab,kw OR demethoxydaunorubicin*:ti,ab,kw OR demethoxydaunomycin*:ti,ab,kw OR mitoxantron*:ti,ab,kw OR ebewe:ti,ab,kw OR mitoxanthron*:ti,ab,kw OR pixantron*:ti,ab,kw OR pixuvri:ti,ab,kw OR daunorubicin*:ti,ab,kw OR cerubidin*:ti,ab,kw OR daunoxome:ti,ab,kw OR leukaemomycin*:ti,ab,kw OR rubidomycin*:ti,ab,kw OR rubomycin*:ti,ab,kw OR daunomycin*:ti,ab,kw OR acetyladriamycin*:ti,ab,kw OR doxorubicin*:ti,ab,kw OR adriamycin*:ti,ab,kw OR caelyx:ti,ab,kw OR adriablastin*:ti,ab,kw OR adriblastin*:ti,ab,kw OR doxil:ti,ab,kw OR myocet:ti,ab,kw OR dactinomycin*:ti,ab,kw OR cosmegen:ti,ab,kw OR actinomycin*:ti,ab,kw OR bleomycin*:ti,ab,kw OR bleomedac:ti,ab,kw OR bleocin*:ti,ab,kw OR bleomicin*:ti,ab,kw OR mitomycin*:ti,ab,kw OR kyowa:ti,ab,kw OR mitocin*:ti,ab,kw OR mitosol:ti,ab,kw OR mitozytrex:ti,ab,kw OR mutamycin*:ti,ab,kw OR mytomycin*:ti,ab,kw OR mytozytrex:ti,ab,kw OR antimetabolite*:ti,ab,kw OR 'anti metabolite*':ti,ab,kw OR purinederivative*:ti,ab,kw OR 'purine derivative*':ti,ab,kw OR 'purine analog*':ti,ab,kw OR 'purine antagonist*':ti,ab,kw OR cladribin*:ti,ab,kw OR leustatin:ti,ab,kw OR chlorodeoxyadenosin*:ti,ab,kw OR litak:ti,ab,kw OR cladarabin*:ti,ab,kw OR clofarabin*:ti,ab,kw OR evoltra:ti,ab,kw OR clolar:ti,ab,kw OR clofarex:ti,ab,kw OR fludarabin*:ti,ab,kw OR fludara:ti,ab,kw OR mercaptopurin*:ti,ab,kw OR xaluprin*:ti,ab,kw OR purinethol*:ti,ab,kw OR merkaptopurin*:ti,ab,kw OR merkaptopuryn*:ti,ab,kw OR hypoxanthin*:ti,ab,kw OR ismipur:ti,ab,kw OR leukerin*:ti,ab,kw OR leupurin*:ti,ab,kw OR mercaleukin*:ti,ab,kw OR purixan:ti,ab,kw OR nelarabin*:ti,ab,kw OR atriance:ti,ab,kw OR arranon:ti,ab,kw OR tioguanin*:ti,ab,kw OR lanvis:ti,ab,kw OR thiosix:ti,ab,kw OR thioguanin*:ti,ab,kw OR 'pyrimidine antagonist*':ti,ab,kw OR 'pyrimidine analog*':ti,ab,kw OR fluorouracil*:ti,ab,kw OR adrucil:ti,ab,kw OR efudix:ti,ab,kw OR arumel:ti,ab,kw OR carac:ti,ab,kw OR carzonal:ti,ab,kw OR fluoruracil*:ti,ab,kw OR azacitidin*:ti,ab,kw OR vidaza:ti,ab,kw OR azacytidin*:ti,ab,kw OR ladakamycin*:ti,ab,kw OR mylosar:ti,ab,kw OR capecitabin*:ti,ab,kw OR xeloda:ti,ab,kw OR xabine:ti,ab,kw OR cytarabin*:ti,ab,kw OR depocyt*:ti,ab,kw OR citarabin*:ti,ab,kw OR alexan:ti,ab,kw OR arabitin*:ti,ab,kw OR aracyt*:ti,ab,kw OR erpalfa:ti,ab,kw OR tarabin*:ti,ab,kw OR udicil:ti,ab,kw OR decitabin*:ti,ab,kw OR dacogen:ti,ab,kw OR gemcitabin*:ti,ab,kw OR zefei:ti,ab,kw OR gemcel:ti,ab,kw OR gemlip:ti,ab,kw OR tegafur:ti,ab,kw OR teysuno:ti,ab,kw OR gimeracil:ti,ab,kw OR oteracil:ti,ab,kw OR tipiracil:ti,ab,kw OR lonsurf:ti,ab,kw OR trifluridin*:ti,ab,kw OR floxuridin*:ti,ab,kw OR deoxyuridin*:ti,ab,kw OR 'alkylating agent*':ti,ab,kw OR busulfan*:ti,ab,kw OR busilvex:ti,ab,kw OR busulfex:ti,ab,kw OR myleran:ti,ab,kw OR bendamustin*:ti,ab,kw OR bendeka:ti,ab,kw OR levact:ti,ab,kw OR carmustin*:ti,ab,kw OR consium:ti,ab,kw OR gliadel:ti,ab,kw OR nitrumon:ti,ab,kw OR chlorambucil*:ti,ab,kw OR leukeran:ti,ab,kw OR clorambucil*:ti,ab,kw OR chlormethin*:ti,ab,kw OR ledaga:ti,ab,kw OR mechlorethamin*:ti,ab,kw OR mustargen:ti,ab,kw OR cyclophosphami*:ti,ab,kw OR endoxan:ti,ab,kw OR cyclofosfami*:ti,ab,kw OR cytophospha*:ti,ab,kw OR dacarbazin*:ti,ab,kw OR 'imidazole carboxamide':ti,ab,kw OR deticene:ti,ab,kw OR iphosphamide:ti,ab,kw OR holoxan:ti,ab,kw OR ifosfami*:ti,ab,kw OR lomustin*:ti,ab,kw OR belustin*:ti,ab,kw OR ceenu:ti,ab,kw OR gleostine:ti,ab,kw OR melphalan*:ti,ab,kw OR alkeran:ti,ab,kw OR melfalan*:ti,ab,kw OR evomela:ti,ab,kw OR

procarbazin*:ti,ab,kw OR natulan:ti,ab,kw OR matulane:ti,ab,kw OR indicarb:ti,ab,kw OR temozolomid*:ti,ab,kw OR temodal:ti,ab,kw OR temodar:ti,ab,kw OR temcad:ti,ab,kw OR methazolastone:ti,ab,kw OR thiotepa:ti,ab,kw OR tepadina:ti,ab,kw OR tiotepa:ti,ab,kw OR treosulfan*:ti,ab,kw OR trecondi:ti,ab,kw OR treosulphan*:ti,ab,kw OR ovastat:ti,ab,kw OR 'microtubule inhibitor*':ti,ab,kw OR taxoid*:ti,ab,kw OR taxan*:ti,ab,kw OR docetax*:ti,ab,kw OR taxotere:ti,ab,kw OR taxoel:ti,ab,kw OR cabazitax*:ti,ab,kw OR jevtana:ti,ab,kw OR paclitaxel:ti,ab,kw OR abraxane:ti,ab,kw OR paclitaxin:ti,ab,kw OR taxol:ti,ab,kw OR capxol:ti,ab,kw OR 'vinca alkaloid*':ti,ab,kw OR vincristin*:ti,ab,kw OR leurocristine:ti,ab,kw OR oncovin*:ti,ab,kw OR vincasar:ti,ab,kw OR citomid:ti,ab,kw OR vinblastin*:ti,ab,kw OR velban:ti,ab,kw OR vincaleukoblastin*:ti,ab,kw OR vincaleucoblastin*:ti,ab,kw OR vinorelbin*:ti,ab,kw OR navelbine:ti,ab,kw OR exelbine:ti,ab,kw OR asparaginase:ti,ab,kw OR erwinase:ti,ab,kw OR spectrila:ti,ab,kw OR elspar:ti,ab,kw OR crisataspase:ti,ab,kw OR colaspase:ti,ab,kw OR cisplatin*:ti,ab,kw OR platinol:ti,ab,kw OR platamin:ti,ab,kw OR neoplatin:ti,ab,kw OR cismaplat:ti,ab,kw OR diamminedichloridoplatin*:ti,ab,kw OR oxaliplatin*:ti,ab,kw OR eloxatin:ti,ab,kw OR oxalisin:ti,ab,kw OR carboplatin*:ti,ab,kw OR carbosin:ti,ab,kw OR paraplatin:ti,ab,kw OR 'topoisomerase inhibitor*':ti,ab,kw OR 'topoisomerase i inhibitor*':ti,ab,kw OR 'topoisomerase ii inhibitor*':ti,ab,kw OR etoposid*:ti,ab,kw OR etopophos:ti,ab,kw OR toposar:ti,ab,kw OR eposin:ti,ab,kw OR toposin:ti,ab,kw OR vepesid:ti,ab,kw OR irinotecan*:ti,ab,kw OR camptosar:ti,ab,kw OR campto:ti,ab,kw OR onivyde:ti,ab,kw OR irinophore:ti,ab,kw OR topotecan*:ti,ab,kw OR hycamtin:ti,ab,kw OR hycamptamine:ti,ab,kw OR nogitecan:ti,ab,kw OR 'arsenic trioxide':ti,ab,kw OR trisenox:ti,ab,kw OR hydroxycarbamid*:ti,ab,kw OR droxia:ti,ab,kw OR hydrea:ti,ab,kw OR hydroxyurea:ti,ab,kw OR carbamazepin*:ti,ab,kw OR methotrexate:ti,ab,kw OR emthexate:ti,ab,kw OR trexall:ti,ab,kw OR rheumatrex:ti,ab,kw OR pegaspargas*:ti,ab,kw OR oncaspar:ti,ab,kw OR realgar:ti,ab,kw OR huangdai:ti,ab,kw OR 'chemotherapy'/exp OR 'regional perfusion'/exp OR 'antineoplastic antibiotic'/exp OR 'antineoplastic antimetabolite'/exp OR 'alkylating agent'/exp OR 'antimitotic agent'/exp OR 'antineoplastic alkaloid'/exp OR 'asparaginase'/exp OR 'dna topoisomerase inhibitor'/exp OR 'arsenic trioxide'/exp OR 'hydroxyurea'/exp OR 'methotrexate'/exp OR 'realgar indigo naturalis formula'/exp) AND (circadian:ti,ab,kw OR clock*:ti,ab,kw OR 'biological rhythm*':ti,ab,kw OR 'chronomodulat*':ti,ab,kw OR 'chrono modulat*':ti,ab,kw OR 'chronotherap*':ti,ab,kw OR 'chrono therap*':ti,ab,kw OR 'chronobiolog*':ti,ab,kw OR 'chrono biolog*':ti,ab,kw OR 'nycthemeral':ti,ab,kw OR nyctohemeral:ti,ab,kw OR diurnal:ti,ab,kw OR chronochemotherap*:ti,ab,kw OR 'chronotherapy'/exp OR 'chronobiology'/exp)) AND [embase]/lim NOT ([embase]/lim AND [medline]/lim) AND ([dutch]/lim OR [english]/lim OR [german]/lim) NOT 'conference abstract'/it
